# Osteocalcin Ameliorates CUMS‐Induced Depressive‐Like Behaviors by Reducing Mitochondrial Damage in Hippocampal Neurons

**DOI:** 10.1111/cns.70530

**Published:** 2025-08-01

**Authors:** Hui Chen, Jindong Mao, Min Wang, Qian Zhang, Rui Zheng, Zhaoxia Zhang, Qianqian Lv, Qingquan Liu, Yumei Wu, Xue Ma

**Affiliations:** ^1^ Department of Pharmacology, School of Pharmacy Fourth Military Medical University Xi'an China; ^2^ Department of Endocrinology, Tangdu Hospital Fourth Military Medical University Xi'an China; ^3^ Department of Metabolism and Endocrinology Xi'an Daxing Hospital Affiliated to Yan'an University Xi'an China

**Keywords:** depression, GPR158, GPR37, hippocampus, mitochondria, osteocalcin, oxidative stress

## Abstract

**Background:**

Depression is a common psychological disorder characterized by limited treatments. Osteocalcin (OCN), a bioactive protein that originates from bone tissue, has been implicated in emotional regulation and the reduction of oxidative stress in peripheral tissues. However, the precise mechanisms by which OCN functions within the central nervous system are still not fully understood.

**Aims:**

This study aimed to clarify the function of OCN in depression‐like behavior, identify its functional brain region, and explore its impact on neuronal mitochondrial function and the exact molecular mechanisms involved.

**Materials and Methods:**

In this study, the antidepressant effects and mitochondrial protective properties of OCN were examined in adult male C57BL/6 mice subjected to chronic unpredictable mild stress (CUMS); then, the potential molecular pathway was explored both in vivo and in vitro conditions. The CUMS model was employed to induce depression in mice. Initially, depressive‐like behaviors in CUMS mice were evaluated following a 3‐week intraperitoneal injection of OCN. Subsequently, the expression levels and distribution of GPR158 and GPR37 were examined. Next, the specific effects of OCN on mitochondrial function were determined. Finally, the molecular pathways through which OCN demonstrates its antidepressant properties and offers mitochondrial protection were explored in both in vivo and in vitro conditions.

**Results:**

OCN significantly alleviated depressive‐like symptoms in CUMS mice, which was evidenced by improvements in weight variations, increased consumption of sucrose, and a greater total distance traveled in the open field test (OFT). Additionally, it shortened the immobility time observed in both the forced swim test and the tail suspension test. OCN influenced hippocampal neuronal activity by modifying the expression levels of PR158 and GPR37, demonstrated by its ability to counteract the downregulation of both receptors in experiments conducted in vivo and in vitro. Furthermore, OCN mitigated mitochondrial damage in neurons induced by depression through the PKA/AMPK/PGC1α signaling pathway, resulting in elevated ATP levels and reduced ROS levels. Notably, inhibiting PKA and AMPK abolished OCN's effects on PGC‐1α, ATP production, and ROS reduction.

**Discussion:**

The administration of OCN significantly ameliorates depressive‐like behaviors in mice, demonstrating the crucial involvement of the bone‐brain pathway in depression pathogenesis and offering further evidence for a better understanding of how peripheral bone tissue affects brain function. The results also provide a novel perspective on the function of OCN in neurons, paving the way for further exploration of innovative therapeutic approaches for central nervous system disorders associated with mitochondrial dysfunction.

**Conclusion:**

Our results indicate that OCN mitigated oxidative stress damage and enhanced mitochondrial function through the AMPK/PGC‐1α pathway, demonstrating antidepressant properties.

## Graphical abstract

After being secreted by osteoblasts, osteocalcin crosses the blood–brain barrier and interacts with GPCR receptors on hippocampal neurons. This interaction triggers a PKA‐dependent activation of the AMPK/PGC1 pathway, which, in turn, promotes the stabilization of mitochondrial function, leading to an improvement in depressive‐like behaviors in mice.

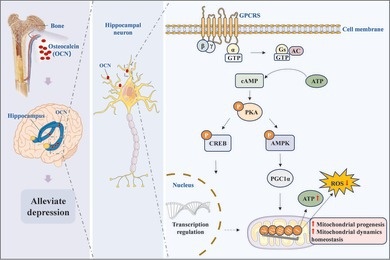

## 1 Introduction

Depression, a prevalent mental health disorder characterized by a persistently low mood state, is closely associated with various factors, including neurotransmitter imbalances, inflammation, genetic predispositions, and gut microbiome alterations. Additionally, chronic stress and mitochondrial dysfunction are recognized as important factors contributing to its onset. Numerous studies indicate that mitochondrial dysfunction in different brain regions is linked to depression, particularly in individuals with moderate to severe major depressive disorder (MDD) and in animal models exposed to chronic stress. This dysfunction leads to reduced adenosine triphosphate (ATP) production, elevated levels of reactive oxygen species (ROS), disrupted mitochondrial dynamics, and structural damage to mitochondria [1, 2, 3, 4]. Mitochondria are essential for neuronal development and maturation, synaptic plasticity, and neurotransmission. Mitochondrial dysfunction can impair energy metabolism and potentially lead to neuronal cell death [5, 6, 7], which is a critical pathological feature of depression and other mitochondrial‐related central nervous system (CNS) disorders [8, 9, 10, 11]. Therefore, enhancing mitochondrial function may provide a promising therapeutic strategy for treating depression.

OCN is a prominent noncollagenous protein primarily produced by osteoblasts. It regulates central nervous system activity by interacting with the G‐protein–coupled receptors GPR158 and GPR37 in the brain [12, 13, 14]. OCN has the ability to cross the blood–brain barrier and can directly bind to neurons in regions like the brainstem, midbrain, and hippocampus, thereby alleviating depression‐like behaviors in mice [13]. An increase in serum OCN has been noted during the treatment of depressed patients [15]. Furthermore, there are notable correlations between serum OCN concentrations and the occurrence of anxiety and depressive symptoms in individuals with primary hyperparathyroidism [16]. These data suggest that OCN may have antidepressant properties, but the exact mechanisms are not fully elucidated.

Although OCN has been reported to increase ATP levels and reduce ROS production in peripheral tissues [17, 18], it is unclear whether OCN exhibits similar antioxidant and mitochondrial protective effects in the CNS and whether these effects contribute to its potential antidepressant actions. Therefore, this study aims to clarify the function of OCN in depression‐like behavior, identify its functional brain region, and explore its impact on neuronal mitochondrial function and exact molecular mechanisms involved. The results of this study are of great significance to reveal the role of the bone–brain pathway in the pathological process of depression and provide a new strategy for the prevention and treatment of depression by OCN.

## 2 Materials and Methods

### 2.1 Reagents and Antibodies

(Glu^13^·^17^·^20^)‐Osteocalcin (1–46) (OCN, 4057685) was purchased from Bachem (Bubendorf, Switzerland). Fluoxetine (BA3590) was purchased from APExBIO (Houston, USA). ATP (A095‐1‐1) and ROS (E004‐1‐1) kits were purchased from Nanjing Jiancheng Bioengineering Institute (Nanjing, China). Fetal bovine serum (FBS), advanced DMEM medium, and penicillin–streptomycin solution (100 ×) were purchased from Gibco (Santa Clara, CA, USA); Trypsin–EDTA solution was purchased from Cytiva (Wilmington, DE, USA); CCK‐8 Kit (E1CK‐000208) was purchased from Enogene (Shanghai, China). Compound C (CoC, S7306) and H89 (S1582) were purchased from Selleckchem (Houston, TX, USA); Antibodies against GPR37 (1:3000, 29,989–1‐AP), PINK1 (1:600, 23,274–1‐AP), AMPK alpha 1 (1:2000, 66,536–1‐Ig), and multi‐rAb HRP‐goat anti‐mouse recombinant secondary antibody (1:10,000, RGAM001) were purchased from Proteintech (Wuhan, China); Antibodies against GPR158 (1:2000, 40,977) were purchased from Signalway (SAB, USA); Antibodies against phosphorylated PKA C (Thr197) (1:2000, CY9083), PGC1α (1:2000, CY6630), phospho‐Creb (Ser133) (1:2000, CY5043), CREB (1:2000, CY5426), Mfn2 (1:1000, CY6638), Drp1 (1:1000, CY8006) were purchased from Abways (Shanghai, China); Antibodies against PKA alpha/beta/gamma CAT (1:2000, AF7746) were purchased from Affinity Biosciences (USA); Antibodies against Iba‐1 (1:1000, ab178846) were purchased from Abcam (UK); Antibodies against phospho‐AMPKα (Thr172) (1:1000, D4D6D), β‐actin (1:10,000, 20,536–1‐AP), GFAP (1:200, 3670S), NeuN (1:200, 94403S) were purchased from Cell Signaling (Danvers, MA, USA); HRP‐goat anti‐rabbit recombinant secondary antibody (1:10000; EK020) and goat anti‐mouse IgG‐FITC (1:200, EK013) were purchased from Xi'an Zhuangzhi (Xi'an, China); Cy3‐conjugated goat anti‐rabbit IgG (1:200, SA00009‐2) was purchased from Proteintech (Wuhan, China).

### 2.2 Animals and OCN Administration

The experiments were carried out in accordance with the Animal Care and Use Committee of the Air Force Medical University (FMMU). Eight‐week‐old male C57BL/6 mice were obtained from FMMU's Laboratory Animal Center and kept under a 12‐h light/dark cycle with unrestricted access to water and food. The mice were randomly divided into six separate groups: One control group (CON), one chronic unpredictable mild stress (CUMS) model group, one CUMS group treated with Fluoxetine (10 mg/kg), and three CUMS treatment groups that received varying doses of OCN (1 μg/kg, 3 μg/kg, and 10 μg/kg).

**FIGURE 1:**
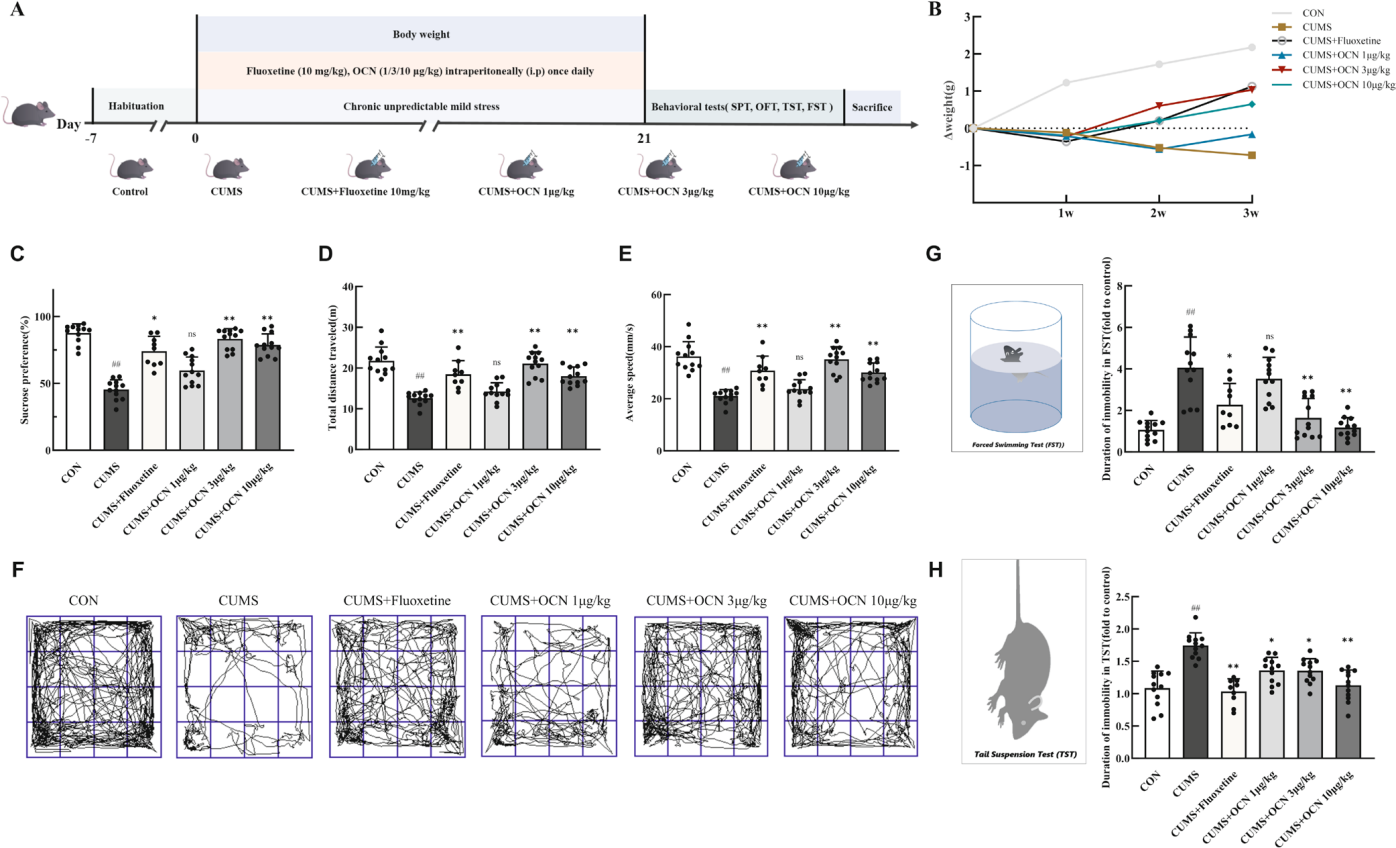
OCN improved depressive behaviors of CUMS mice. Mice were injected intraperitoneally with either fluoxetine (10 mg/kg) as a positive control or various doses of OCN (1, 3, or 10 μg/kg) for 3 weeks, followed by behavioral tests. (A) Experimental arrangement and animal grouping. (B) Weight alterations of mice. (C) Percentage of sucrose preference (%). (D, E) Total distance traveled and mean speed in the OFT. (F) Representative paths observed during the OFT. The fold‐change relationship with the immobility time of the control group mice in the FST (G) and TST (H). *Note: n* = 9–12, results are expressed as means ± SEM, # compared to control group; */ns compared to CUMS group, */# p < 0.05, **/## p < 0.01, ***/###, p < 0.001. Shapiro–Wilk test was used to assess data normality; Kruskal–Wallis test followed by Dunn's test for post hoc analysis in SPT; One‐way ANOVA followed by Tukey's post hoc test in OFT, FST, and TST. Abbreviations: CUMS, chronic unpredictable mild stress; FST, forced swimming test; OCN, osteocalcin; OFT, open field test; SPT, sucrose preference test; TST, tail suspension test.

Considering that peripheral delivery is consistent with feasible human administration routes and repeated hippocampal procedures may introduce confounding stress in our CUMS paradigm, an approach supported by previous studies [19, 20], we chose intraperitoneal (i.p.) injection to demonstrate the systemic efficacy of OCN and Fluoxetine. This selection was made to better reflect the potential for clinical translation and to preserve the validity of the model. Fluoxetine and OCN were diluted in sterile water and given intraperitoneally at the designated doses once daily for 3 weeks. Body weight measurements were taken on Days 0, 7, 14, and 21, followed by behavioral tests aimed at evaluating depressive‐like behaviors, as illustrated in Figure 1A.

### 2.3 Chronic Unpredictable Mild Stress Model

To create a model of depression in mice, the CUMS approach was employed, involving exposure to a variety of stressors over a duration of 3 weeks. This approach was slightly adjusted from previous methods [21, 22]. The stressors included reversed light/dark cycles, 24‐h deprivation of food or water, 6 h of overcrowding, 24‐h tilted cage housing, 30 min of cage shaking, 24‐h exposure to moist bedding, 1 h of restraint, and 1 min of tail clamping, all delivered in a nonsequential and unpredictable manner.

### 2.4 Behavioral Tests

#### 2.4.1 The Sucrose Preference Test (SPT)

The SPT is a valuable technique for evaluating anhedonia symptoms and the level of depression in animals. Solutions with a 1%–2% sucrose concentration are commonly employed to determine whether mice experience anhedonia and depression [23, 24, 25]. Since C57BL/6J mice had comparable intake of 1% sucrose solution and water, but significant differences arose at 2% sucrose concentration or above [26]. So, we selected a 2% sucrose solution to both distinguish depressive‐like behavior and reduce animal use. Before formal testing begins, mice are housed individually and undergo a training phase to adapt to sucrose. During this phase, they are given two bottles filled with sucrose water (2% sucrose, 100 mL) for a period of 12 h. Subsequently, one bottle is replaced with regular water for another 12 h, after which the positions of the bottles are swapped to eliminate any preference based on location. Following the adaptation phase, the mice experience a deprivation period of water and food for 12 h. Subsequently, preweighed bottles containing sucrose water and plain water are positioned in their assigned locations within the cages. After another 24 h, the bottles are collected, weighed, and the amounts of sucrose water and regular water consumed by the mice are measured. These data were utilized to calculate the sucrose preference rate using the following formula: Sucrose Preference Rate (%) = (Consumption of sucrose water (g)/Total liquid consumption (g)) × 100%.

#### 2.4.2 Open‐Field Test (OFT)

Mice were placed in a 30 × 30 cm open field, the floor of which was divided into 16 equal squares, with the central four squares designated as the central zone. For a duration of 10 min, mice were allowed to explore freely. The speed and distance moved were subsequently analyzed.

#### 2.4.3 Forced Swimming Test (FST)

Each mouse was placed individually into a cylindrical vessel, which measured 20 cm in height and 15 cm in diameter. The cylindrical vessel was filled with water, and the water temperature was maintained at 23°C–25°C. The swimming activity of the mice was observed for 6 min, and the time spent immobile was noted from the 2nd to the 6th minute.

#### 2.4.4 Tail Suspension Test (TST)

The mice were securely fastened in an inverted position to a hook for a period of 6 min. The last 4 min was recorded to document the duration of immobility.

### 2.5 Western Blot Analysis

HT‐22 cells and hippocampal tissue were lysed in ice‐cold RIPA buffer containing a 1 × mixture of protease and phosphatase inhibitors to isolate total protein. After lysis, samples were centrifuged at 12,000 rpm for 20 min at 4°C, and the resulting supernatants were collected for protein measurement. Equal quantities of protein were subjected to SDS‐PAGE and then transferred to polyvinylidene fluoride membranes. For blocking, the membranes were treated with a 5% nonfat milk solution in 0.1% PBST at room temperature for 2 h, after which they were incubated overnight at 4°C with the primary antibody. After performing three washes with PBST, the membranes were treated with HRP‐conjugated secondary antibody at room temperature for 1 h, and detection was performed using an electrochemiluminescence (ECL) reagent. Finally, protein bands were visualized using the ChemiDoc XRS (Bio‐Rad, Hercules, CA, USA) and quantified with ImageJ software.

### 2.6 Immunofluorescence (IF)

Mice were anesthetized and subsequently perfused with 0.9% saline solution, followed by a perfusion using 4% paraformaldehyde (PFA). Brains were then carefully dissected and postfixed in 4% PFA for 6 h. The tissue was dehydrated through successive immersions in 20% and 30% sucrose solutions and subsequently embedded in OCT compound. Coronal sections of 20 μm thickness were cut. For immunofluorescence analysis, a solution containing 0.3% Triton X‐100 and goat serum was used to block the sections for 1 h at room temperature. Sections were then incubated overnight at 4°C with primary antibodies against GPR37 (1:100), GPR158 (1:100), Iba‐1 (1:200), GFAP (1:200), and NeuN (1:200). After three washes in PBS, the sections were incubated with secondary antibodies for 2 h in the dark at room temperature. Following an additional three washes with PBS, the sections were mounted in 70% glycerol and then observed using a fluorescence microscope.

### 2.7 Transmission Electron Microscopy (TEM)

Following deep anesthesia, a 0.9% normal saline and 4% paraformaldehyde solution was used to perfuse the mice. Subsequently, a block of hippocampal tissue, approximately 1 mm^3^ in size, was quickly excised. The tissue was preserved in fixative for 2 h at 4°C, followed by treatment with 1% osmium tetroxide. After staining with uranyl acetate, the tissue underwent dehydration and was embedded in epoxy resin. Ultrathin sections, measuring between 60 and 80 nm in thickness, were subsequently prepared. Finally, the morphology of the mitochondria was observed using a transmission electron microscope (HT7800, manufactured by HITACHI in Tokyo, Japan) after staining with lead citrate.

### 2.8 Reactive Oxygen Species (ROS) Detection

Hippocampal tissue from mice was rinsed with prechilled 0.01 M PBS according to the ROS assay kit protocol. The tissue was then digested in a 0.25% trypsin solution for 20 min at 37°C in a water bath. Following digestion, the cell suspension was filtered through a 300‐mesh nylon filter and centrifuged at 500*g* for 10 min to concentrate the cells. The cells were washed twice with PBS, then resuspended in PBS that contained 10 μM DCFH‐DA, and incubated at 37°C for 40 min. After incubation, the cells were subjected to a second centrifugation at 1000*g* for 10 min; the supernatant was discarded, and the cells were washed twice more with PBS. Finally, the cells were resuspended in PBS, and the fluorescence intensity of DCF was measured using the FITC channel on a flow cytometer. The methodology for quantifying ROS levels in HT‐22 cells was identical to that used for the cells extracted from mouse hippocampal tissue. Flow cytometry data were analyzed utilizing a Coulter XL flow cytometer (Beckman Coulter, Brea, CA, USA).

### 2.9 Enzyme‐Linked Immunosorbent Assay (ELISA)

To determine ATP concentration, ATP ELISA kits for mice were used following the manufacturer's instructions. Hippocampal tissue and HTT‐22 cells were collected for further analysis.

### 2.10 Cell Culture and Cell Viability Detection

The HT‐22 cell line was derived from the mouse hippocampal neuron and obtained from Dr. Yongli Jiang. It was maintained in DMEM enriched with 10% fetal bovine serum alongside 100 U/mL of penicillin and 100 μg/mL of streptomycin. These cells were maintained in an environment of 5% CO_2_ at a temperature of 37°C. To assess cell viability, 2 × 10^4^ cells were seeded into each well of a 96‐well plate. Subsequently, the cells were exposed to varying concentrations of OCN (1, 3, 10, 30, 100 nM) or corticosterone (CORT 50, 100, 200, 300, 400, 500 μM); each well received 10 μL of CCK‐8 solution and was incubated for 50 min. The results were then measured using a microplate reader at 450 nm, with data expressed as a percentage of the control group.

### 2.11 Statistical Analysis

Statistical analyses for this research were performed using GraphPad Prism 8.0 software. To assess differences among the groups, we first used the Shapiro–Wilk test to check whether the sample data of each group followed a normal distribution. If the data met the normality criterion, we conducted either one‐way or two‐way ANOVA, followed by Tukey's post hoc test. Otherwise, we applied the Kruskal–Wallis test and used Dunn's test for post hoc analysis. Results from each experiment are presented as mean values with corresponding standard deviations (SD). A *p* value of less than 0.05 was considered statistically significant.

## 3 Results

### 3.1 Osteocalcin Ameliorates Depressive‐Like Behaviors in CUMS Mice

Recent research indicates that mice lacking OCN (OCN^−/−^) display behaviors associated with anxiety and depression, while intracerebroventricular (ICV) injection of OCN effectively reverses these behaviors in mice [13]. However, it remains to be determined whether OCN can directly alleviate depressive symptoms in established depression models. We, therefore, first evaluated the antidepressant effects of OCN in mice exposed to CUMS. The mice were administered intraperitoneal injections of 1 μg/kg, 3 μg/kg, or 10 μg/kg OCN once daily for 3 weeks, and fluoxetine (10 mg/kg) was intraperitoneally injected into CUMS mice to serve as a positive control group, as shown in Figure 1A. Body weight changes as a result of exposure to psychosocial stressors, so monitoring weight can be used to assess overall health in response to mental stress. We observed a progressive decline in body weight among CUMS mice over 3 weeks compared to control mice, resulting in a reduction in body weight. Treatment with three doses of OCN alleviated the weight loss of CUMS mice to varying degrees. Among them, the effect of OCN at a dose of 3 μg/kg was comparable to that of fluoxetine (Figure 1B). Additionally, we utilized the sucrose preference test to assess depressive behavior. CUMS mice exhibited a significantly reduced sucrose preference compared to wild‐type (WT) mice. Notably, fluoxetine and OCN (3 μg/kg and 10 μg/kg OCN) treatment restored sucrose consumption in the CUMS mice (Figure 1C). During the 10‐min OFT, both the total distance traveled and movement speed of CUMS mice were significantly reduced compared to WT mice. In contrast to the CUMS group, the administration of 3 μg/kg and 10 μg/kg OCN significantly improved these parameters, and the effect was comparable to that of fluoxetine (Figure 1D–F). The FST and TST are widely employed to evaluate potential antidepressant effects. Results from the FST (Figure 1G) and TST (Figure 1H) showed that the immobility duration in CUMS mice was significantly prolonged compared to control mice; however, this increase was reversed by OCN treatment at both 3 μg/kg and 10 μg/kg doses; moreover, these two doses of OCN had an effect similar to that of fluoxetine. These findings indicate that both 3 μg/kg and 10 μg/kg OCN effectively ameliorate depressive behaviors in CUMS mice. Since a dose of 3 μg/kg of OCN has already demonstrated a stable effect in improving depressive‐like behaviors, we selected 3 μg/kg OCN for subsequent experiments.

### 3.2 Osteocalcin Restored the Expression Levels of GPR158 and GPR37 in Hippocampal Neurons

**FIGURE 2:**
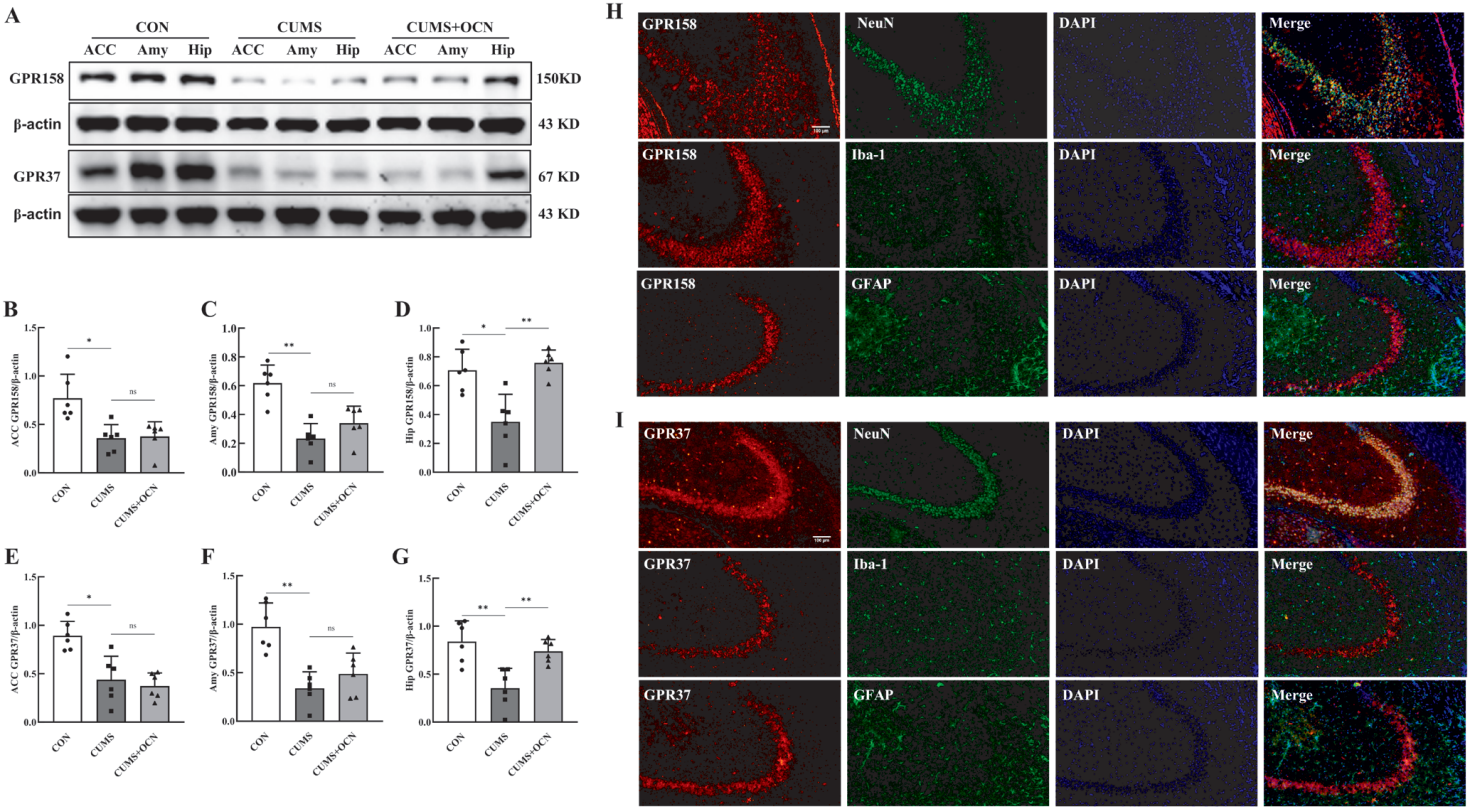
OCN facilitated the recovery of GPR158 and GPR37 expression levels in the hippocampus of CUMS mice. (A) Representative Western blot protein bands of GPR158 and GPR37. The protein levels of GPR158 in the ACC (B), Amy (C), and Hip (D) of the mouse brain. The protein levels of GPR37 in the ACC (E), Amy (F), and Hip (G) of the mouse brain (*n* = 6). Representative images illustrating GPR158 (H) and GPR37 (I) in neurons, microglia, and astrocytes within the hippocampus of CUMS mice treated with OCN. GPR158 and GPR37 are visualized as red; Neurons were identified using NeuN (green), microglia were stained with Iba‐1 (green), and GFAP (green) was employed to label astrocytes, while nuclei were visualized using DAPI (blue). Scale bar, 100 μm. The data are presented as mean ± SEM. Note: *p < 0.05, **p < 0.01. Shapiro–Wilk test to assess data normality, followed by one‐way ANOVA with Tukey's post hoc test. Abbreviations: Acc: Anterior cingulate cortex; Amy: Amygdala; Hip: Hippocampus.

To identify the central target of OCN's antidepressant effects, we investigated the expression and distribution of GPR158 and GPR37 receptors in mouse brain. We initially assessed the expression levels of these two receptors in brain regions associated with depression. Western blot analyses demonstrated a reduction in GPR158 and GPR37 expression within the anterior cingulate cortex (Acc), amygdala (Amy), and hippocampus (Hip) in CUMS mice, when compared to control mice. Notably, treatment with OCN considerably restored levels of these receptors in the hippocampus, but no such changes were detected in the ACC and Amy regions (Figure 2A–G). These results implied that the effect of OCN on reducing depressive‐like behaviors caused by CUMS in mice could be associated with its function in the hippocampus. Therefore, the role of OCN in the hippocampus should be further studied.

To clarify the cellular selectivity of OCN, we identified the cell types expressing GPR158 and GPR37 in CUMS mice by immunofluorescence in the mouse hippocampus, and colabeled the two receptors with the neuronal marker NeuN, the astrocyte marker GFAP, and the microglial marker Iba‐1 (Figure 2H,I). The results showed that GPR158 and GPR37 are primarily found on neurons in the mouse hippocampus, but were not significantly expressed in astrocytes or microglia, suggesting that OCN may regulate the behavior of CUMS mice by directly affecting neuronal function.

### 3.3 Osteocalcin Alleviated Mitochondrial Dysfunction in the Hippocampus

**FIGURE 3:**
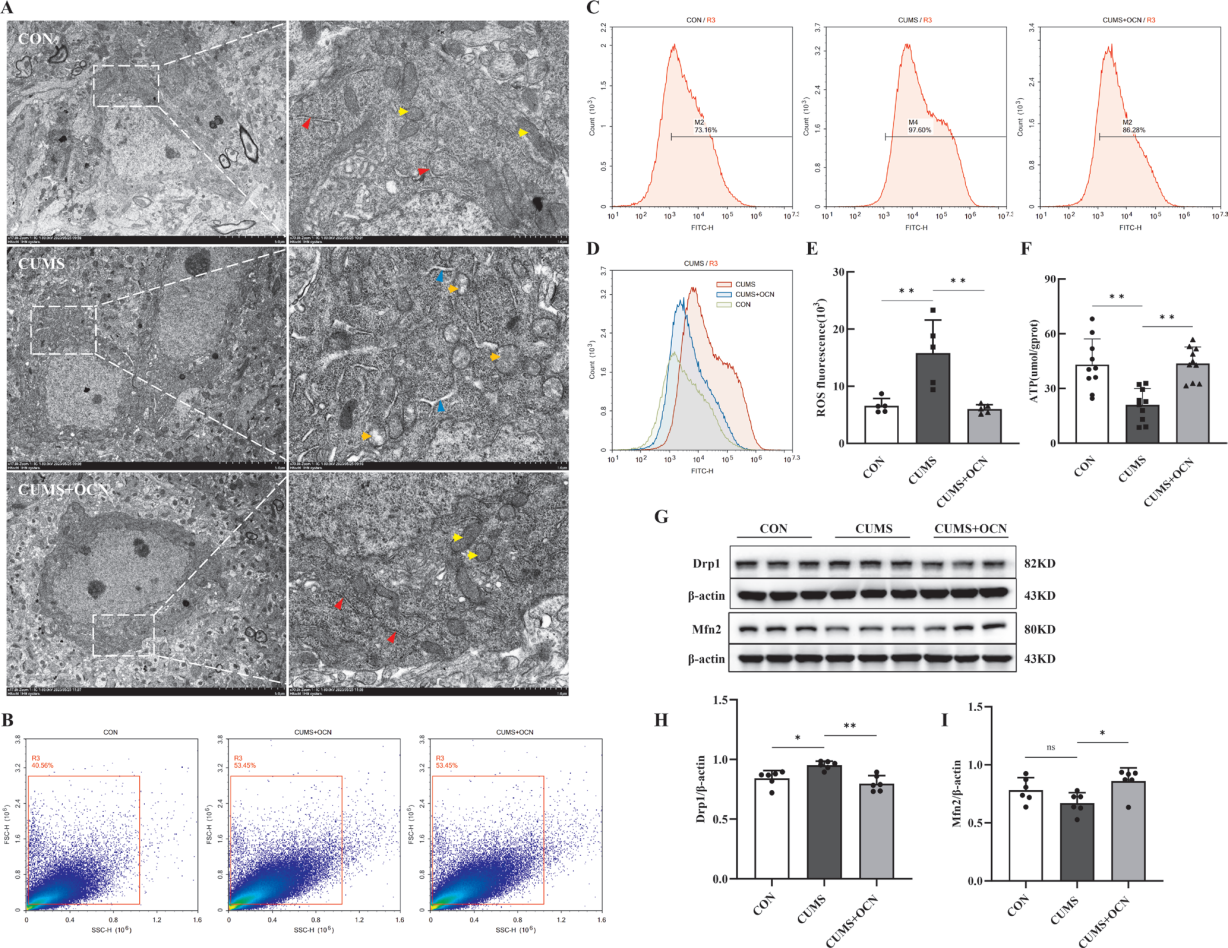
OCN alleviated CUMS‐induced mitochondrial damage in hippocampal neurons. After the behavioral detection was completed, the hippocampal tissues of mice were extracted for the detection of mitochondria morphology and function. (A) TEM images of mitochondria and endoplasmic reticulum in hippocampal neurons. Yellow arrow, healthy mitochondria; Orange arrow, damaged mitochondria; Red triangle, healthy endoplasmic reticulum; Blue triangle, expanded endoplasmic reticulum; Scale bar, left (5 μm), right (1 μm). (B–E) The level of ROS in hippocampus was assessed through DCFH‐DA staining, *n* = 5. (F) The quantification of ATP in hippocampus was determined using ELISA assay, *n* = 10. (G) Representative Western blot protein bands of Drp1 and Mfn2 in mice hippocampal tissue. The protein levels of Drp1 (H) and Mfn2 (I), *n* = 6, Note: **p < 0.05, **p < 0.01. Shapiro–Wilk test to assess data normality, followed by one‐way ANOVA with Tukey's post hoc test.

To assess the influence of OCN on the mitochondria within hippocampal neurons, we analyzed mitochondrial structure using transmission electron microscopy. Our study found that the usual shape of mitochondria in healthy mice was predominantly rounded or elliptical (Figure 3A, yellow arrow). However, in mice subjected to CUMS, mitochondria exhibited a specialized morphology that included spherical expansion, increased brightness, and vacuole‐like alterations (Figure 3A, orange arrow). Nonetheless, OCN administration normalized mitochondrial structure in CUMS mice. Furthermore, an expansion of the endoplasmic reticulum was observed in the hippocampal neurons of the CUMS group (Figure 3A, blue triangle), whereas the morphology of the endoplasmic reticulum in the CUMS + OCN group resembled that of the control group, displaying normal features (Figure 3A, red triangle). These findings indicated that OCN alleviated mitochondrial damage and endoplasmic reticulum stress in hippocampal neurons of depressed mice.

Sustained exposure to mild chronic stress can impair mitochondrial complexes I, III, IV, and IV, resulting in reduced ATP production and elevated ROS levels [4, 27, 28, 29]. Chronic stress can also disrupt mitochondrial dynamics. To investigate the precise influence of OCN on mitochondrial function, we first measured the concentrations of ROS and ATP in the hippocampal tissue of mice. Our findings revealed that mice subjected to CUMS displayed higher ROS levels (Figure 3B–E) and lower ATP levels (Figure 3F) compared to WT mice. Importantly, the treatment of OCN restored the levels of both ATP and ROS in the hippocampus of CUMS mice. Mitochondrial fusion and fission are critical mechanisms for maintaining mitochondrial homeostasis; we observed increased expression of dynamin‐related protein 1 (Drp1) (Figure 3G,H) and decreased expression of mitofusin 2 (Mfn2) (Figure 3G,I) in hippocampal tissues of CUMS mice; treatment with OCN restored their expression.

### 3.4 Osteocalcin Ameliorated Mitochondrial Dysfunction Induced by CUMS Through the AMPK/PGC1α Signaling Pathway

**FIGURE 4:**
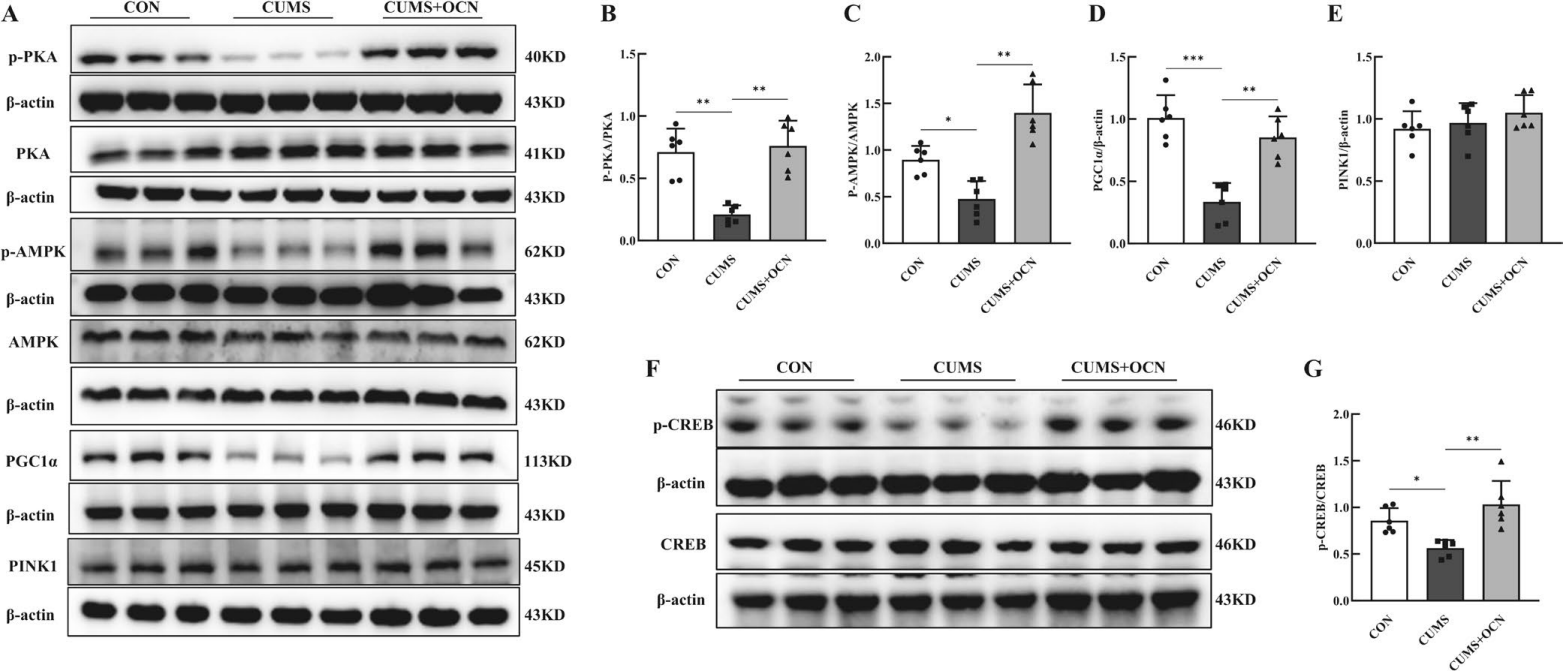
The AMPK/PGC1α signaling pathway mediated the effects of OCN in the mouse hippocampus. Representative Western blot bands (A) and quantitative analyses (B–E) of p‐PKA, PKA, p‐AMPK, AMPK, PGC1α, and PINK1 in mice hippocampal tissue. (F–G) Representative Western blot analyses of p‐CREB and CREB in the hippocampus. *Note: n* = 6, *p < 0.05, **p < 0.01. ***p < 0.001. Shapiro–Wilk test to assess data normality, followed by one‐way ANOVA with Tukey's post hoc test.

AMP‐activated protein kinase (AMPK) acts as a key regulator of mitochondrial activities, including fusion, fission, and biogenesis [30, 31, 32]. The PKA/AMPK signaling pathway plays a critical role in modulating mitochondrial fission [33]. To elucidate the molecular mechanism of OCN, we investigated whether OCN alleviates mitochondrial damage through the PKA/AMPK signaling pathway. Western blot analysis of hippocampal tissue showed a significant decrease in levels of phosphorylated PKA (p‐PKA) and phosphorylated AMPK (p‐AMPK) in mice subjected to CUMS when compared to control subjects. In contrast, OCN administration led to a notable rise in p‐PKA and p‐AMPK levels in CUMS mice, but there was no significant difference in the expression levels of PKA and AMPK total protein (Figure 4A–C). PGC1α is an important downstream target of AMPK, significantly contributing to the preservation of normal mitochondrial function and the process of biogenesis [34, 35, 36]. We further evaluated the levels of PGC1α expression in the hippocampus. The PGC1α protein levels were markedly lower in CUMS mice compared to their WT counterparts (Figure 4A,D). Importantly, OCN administration rescued the decrease in PGC1α expression. However, OCN had no effect on the expression of PINK1, an important mitophagy marker (Figure 4A,E). These results suggest that OCN treatment ameliorates mitochondrial damage through the AMPK/PGC1α‐dependent pathway, highlighting the significance of the AMPK/PGC1α signaling pathway in mediating the effects of OCN on mitochondrial function.

PKA and its downstream substrate proteins, like cAMP response element‐binding protein (CREB), may have an effect on gene transcription [37]. Previous research has found that CREB is involved in the downstream signaling of OCN in PTEN‐null human prostate cancer cell lines [38], and we also evaluated the expression changes of CREB in the hippocampus. We detected a decrease in phosphorylated CREB (p‐CREB) levels in CUMS mice, while OCN treatment significantly increased p‐CREB levels (Figure 4F,G).

### 3.5 Osteocalcin Enhanced the Growth of HT‐22 Cells and Alleviated the Damage Caused by Corticosterone

**FIGURE 5:**
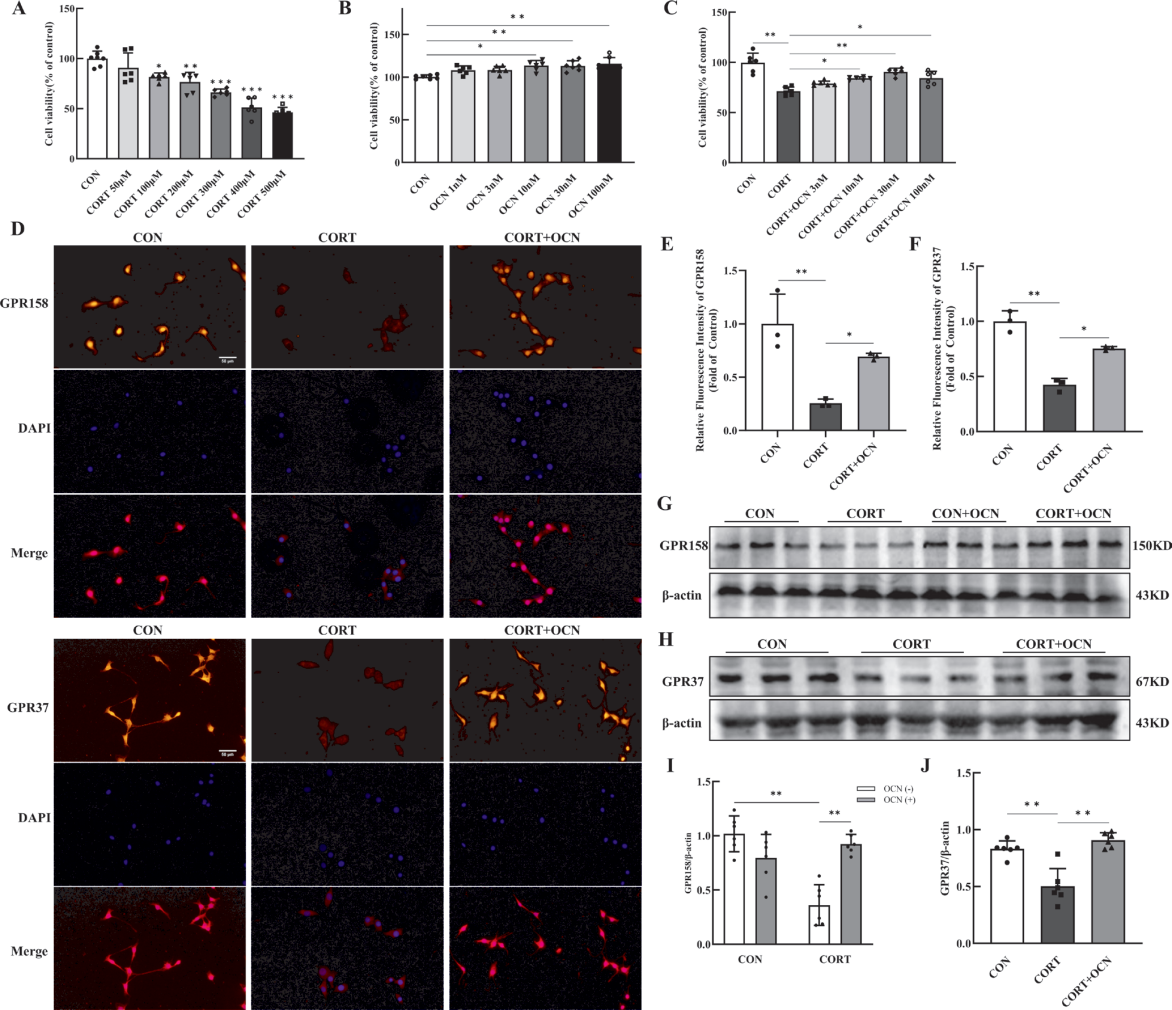
OCN reduced the HT‐22 cells damages and reversed the CORT‐induced downregulation of GPR158 and GPR37. (A) The viability of HT‐22 cells subjected to various concentrations of CORT (50, 100, 200, 300, 400, and 500 μM) for 24 h (*p < 0.05, **p < 0.01, ***p < 0.001 vs. CON). (B) The viability of HT‐22 cells cultured with various concentrations of OCN (1, 3, 10, 30, and 100 nM) for 24 h. (C) The viability of HT‐22 cells treated with various concentrations of OCN (3, 10, 30, and 100 nm) for 2 h prior to the 24‐h CORT treatment. After being pretreated with OCN (30 nM) for 2 h, the cells were incubated with CORT (200 μM) for 24 h, and then the expression of GPR158 and GPR37 were observed via immunofluorescence staining and Western blots. (D) Representative staining images of GPR158 and Gpr37 in HT‐22 cells, scale bar, 50 μm. The fluorescence intensity of GPR158 (E) and Gpr37 (F). Representative Western blots protein bands of GPR158 (G) and Gpr37 (H) in HT‐22 cells. (I, J) Quantitative analyses of GPR158 and Gpr37. Shapiro–Wilk test to assess data normality, followed by one‐way (A–F, J) and two‐way (I) ANOVA with Tukey's post hoc test, data are shown as mean ± SEM based on a minimum of three separate experiments. Note: *p < 0.05, **p < 0.01, ***p < 0.001. Abbreviation: CORT, corticosterone.

We further used in vitro experiments to elucidate the molecular mechanism of OCN to enhance mitochondrial function and play an antidepressant role. The cultured HT‐22 hippocampal neurons were exposed to CORT to establish a depression cell model. We firstly identified the optimal concentration of CORT and OCN for HT‐22 cell treatment. Various concentrations of CORT (50, 100, 200, 300, 400, and 500 μM) were applied over a 24‐h period, while OCN was evaluated at concentrations of 1, 3, 10, 30, and 100 nM for the same duration. Cell viability was then assessed by CCK‐8 assays. The results indicated that CORT concentrations between 100 μM and 400 μM significantly reduced the viability of HT‐22 cells (Figure 5A); the application of OCN at concentrations ranging from 10 nM to 100 nM led to a dose‐dependent enhancement of cell proliferation (Figure 5B).

Given that a notable reduction in cell viability was detected at 200 μM CORT, we chose this concentration for our following experiments. We next determined the optimal concentration of OCN for treating HT‐22 cells with 200 μM CORT. HT‐22 cells were pretreated with varying concentrations of OCN for 2 h and then exposed to 200 μM CORT for 24 h. The results demonstrated that OCN concentrations between 10 nM and 100 nM effectively reduced the damage induced by CORT, with the 30 nM dose exhibiting particularly pronounced efficacy (Figure 5C). Therefore, we chose the 30 nM concentration of OCN for further experimental studies.

### 3.6 Osteocalcin Rescued the Decrease in GPR158 and GPR37 Levels Induced by Corticosterone in HT‐22 Cells

To investigate the function of OCN in hippocampal neurons, we evaluated how OCN influences the expression of GPR158 and GPR37 in HT‐22 cells. Results from immunofluorescence staining showed that the expression trends of GPR158 and GPR37 in HT‐22 cells were consistent with our previous in vivo findings. After a 24‐h exposure to CORT, a notable decrease in the levels of GPR158 and GPR37 was noted in the HT‐22 cells. OCN treatment restored the expression levels of both receptors (Figure 5D–F), which was consistent with Western blot analysis (Figure 5G–J).

### 3.7 Osteocalcin Mitigated Corticosterone‐Induced Mitochondrial Damage in HT‐22 Cells via the AMPK/PGC1α Pathway

**FIGURE 6:**
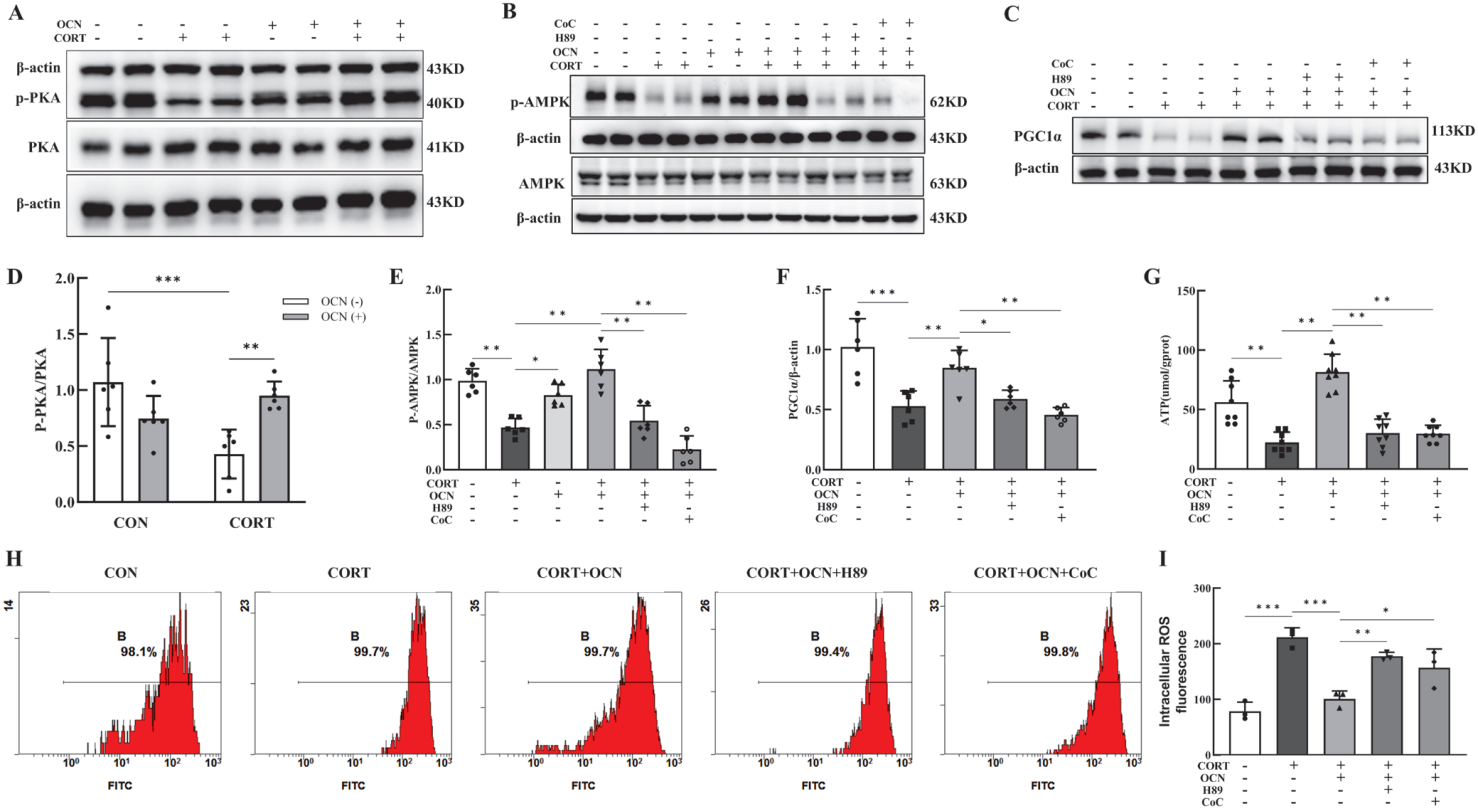
OCN alleviated mitochondrial impairment induced by CORT in HT‐22 cells through the AMPK/PGC1α signaling pathway. HT‐22 cells were pre‐treated with either 15 μM H89 or 15 μM CoC for 2 h, followed by exposure to 30 nM OCN for an additional 2 h, and then treated with CORT for another 2 h. Finally, the cells were collected for subsequent experiments. (A–C) Western blot representative bands of PKA, p‐PKA, p‐AMPK, AMPK, and PGC1α in HT‐22 cells. (D–F) Quantitative analysis of PKA, p‐PKA, p‐AMPK, AMPK, and PGC1α levels, *n* = 6. (G) Intracellular ATP quantification. (H, I) Intracellular ROS fluorescence intensity. Shapiro–Wilk test to assess data normality, followed by two‐way (D) and one‐way (E‐I) ANOVA with Tukey's post hoc test. The date are presented as mean ± SEM from at least three independent experiments. Note: *p < 0.05, **p < 0.01, ***p < 0.001.

We further validated the significance of the AMPK/PGC1α signaling pathway in the action of OCN in HT‐22 cells. HT‐22 cells were either treated with OCN or left untreated for a duration of 2 h before exposure to 200 μM CORT for an additional 2‐h period. The expression levels of p‐PKA (Figure 6A,D), p‐AMPK (Figure 6B,E), and PGC1α (Figure 6C,F) were lower in cells that only received CORT treatment when compared to the control group. OCN pretreatment significantly enhanced the expression of these proteins, aligning with findings obtained in vivo.

H89 is a selective and potent PKA inhibitor; Compound C (CoC) is widely used to inhibit AMPK. We used H89 and CoC to verify the involvement of the AMPK/PGC1α signaling pathway in the role of OCN. We observed that H89 significantly reduced AMPK phosphorylation levels (Figure 6B), indicating the requirement of PKA activation for AMPK phosphorylation. Furthermore, the elevation of PGC1α expression in cells treated with OCN was inhibited by both CoC and H89 (Figure 6C), suggesting the involvement of PKA and AMPK in the regulation of PGC1α expression. Additionally, we investigated how these inhibitors influence mitochondrial function in HT‐22 cells. It was found that after exposure to CORT alone, ATP levels decreased markedly (Figure 6G) and ROS levels increased significantly (Figure 6H,I) compared to controls. Conversely, pretreatment with H89 or CoC blocked the protective effects of OCN, resulting in no significant differences in ATP and ROS levels in the CORT + OCN + H89 and CORT + OCN + CoC groups relative to the CORT + OCN group. These results indicate that OCN treatment protects mitochondrial damage through the AMPK/PGC1α‐dependent pathway.

## 4 Discussion

A variety of regulatory molecules synthesized and secreted by bone tissue can enter the blood, cross the blood–brain barrier, and affect the function of the brain. OCN secreted by osteoblasts significantly contributes to brain development, emotion regulation, and neurocognitive function [13, 39]. After entering the brain, OCN can increase the production and release of norepinephrine by interacting with GPR158 on neurons in the hippocampal CA3 region, midbrain's ventral tegmental area (VTA), and the brainstem's dorsal raphe nucleus (DRN). Additionally, it can stimulate the expression of brain‐derived neurotrophic factor (BDNF), which supports the growth, survival, and synaptic plasticity of neurons [13, 40]. This ultimately aids in spatial learning and memory, improves cognitive function, and alleviates anxiety, depressive behaviors, and age‐related memory loss [13, 39]. Recent studies have confirmed that GPR37 is also the receptor of OCN and mediates its function in oligodendrocytes. OCN regulates oligodendrocyte differentiation and myelination in the central nervous system through GPR37 [14, 41]. In our study, we found that the expression levels of GPR158 and GPR37 were significantly changed in CUMS mice with depression‐like behaviors. OCN appears to regulate the expression of these receptors, suggesting that its positive effects on depression‐like behaviors may be mediated by GPR158 and GPR37, implying that these receptors could be involved in the regulation of OCN on emotion. However, a limitation of the current study is the lack of experiments using specific antagonists for GPR158 and GPR37 to directly validate their functional role in OCN‐mediated effects. This is primarily due to the current lack of widely available, commercially sourced antagonists for these receptors, with few validated options documented in existing literature [40, 42, 43, 44, 45, 46]. The use of nonspecific antagonists was deemed inappropriate here, as it would introduce off‐target effects and complicate the precise attribution of OCN's actions to GPR158 or GPR37 signaling. To address this gap in future investigations, we aim to implement genetic strategies—including targeted knockdown and knockout models of GPR158 and GPR37. Such approaches will enable us to dissect the specific contributions of each receptor to OCN‐mediated effects on AMPK/PGC1α activation, mitochondrial homeostasis, and behavioral responses. These studies will be critical to establishing the causal role of GPR158 and GPR37 in OCN's neuroprotective mechanisms, thereby strengthening the translational relevance of our current observations.

The selection of the hippocampus as the key target region for OCN is further supported by its role in emotional regulation and stress response. As a brain region highly susceptible to chronic stress‐induced damage, the hippocampus exhibits prominent mitochondrial dysfunction and neuronal atrophy in depression models, which aligns with the core pathological features addressed in our study [1, 2, 3, 47]. Additionally, the hippocampus serves as a critical hub for emotional memory processing, and its functional impairment is closely linked to the pathogenesis of depression and anxiety disorders [48, 49, 50]. Our findings demonstrating that OCN specifically restores GPR158 and GPR37 expression in the hippocampus—compared to other emotion‐related brain regions—further validate its selectivity as a target. This receptor‐mediated specificity, combined with the hippocampus's neurobiological significance in mood regulation and the documented ability of OCN to cross the blood–brain barrier and act on hippocampal neurons [13], collectively justifies our focus on this region. Therefore, we selected the hippocampal brain region to further explore the mechanism of action of OCN.

Mitochondria play a crucial role in brain function by generating ATP, which is vital for brain cells. A lack of ATP can result in the death of these cells. During ATP production, ROS are generated as harmful byproducts alongside the respiration process. Under normal conditions, ROS levels are kept at a manageable steady state, which supports proper redox signaling in brain cells [51]. However, an overproduction of ROS can lead to the oxidation of brain lipids and neurotransmitters, resulting in an increase in unsaturated lipids, auto‐oxidation of neurotransmitters, and RNA oxidation, among other effects [52, 53]. Notably, mitochondria also contain antioxidant enzymes and natural antioxidants that help maintain a balance between oxidation and reduction within the cells [54, 55]. Oxidative stress is closely associated with depression, which is characterized by a significant elevation in biomarkers such as ROS, lipid peroxides, and oxidized glutathione [56, 57]. Notably, the overproduction of ROS due to oxidative stress can cause structural and functional damage to mitochondria, leading to a harmful cycle that exacerbates oxidative stress. Our findings demonstrated that OCN significantly reduced ROS content in the hippocampus of CUMS mice and in CORT‐treated HT‐22 cells. It also enhanced ATP production, alleviated mitochondrial swelling, and preserved mitochondrial dynamics through activation of the AMPK/PGC1α signaling pathway. These findings indicate that OCN may play a protective role against oxidative stress and mitochondrial dysfunction.

AMPK is a sensor and metabolic regulator of cellular energy status. It activates or inhibits downstream signaling pathways by sensing changes in the intracellular AMP/ATP ratio, subsequently regulating the metabolic state of cells [58]. Due to the activation of the hypothalamic pituitary adrenal (HPA) axis induced by chronic stress, elevated corticosterone levels and inhibition of AMPK activity are often observed in depression [59, 60, 61]. This is consistent with our finding that the levels of p‐AMPK and PGC1α are reduced in the hippocampus of mice exposed to chronic unpredictable mild stress.

AMPK regulates mitochondrial biogenesis by directly phosphorylating and activating its downstream targets, such as PGC‐1α and SIRT1 [58, 62, 63]. PGC‐1α plays a crucial role in maintaining the functionality of mitochondria. It facilitates the replication and transcription of mitochondrial DNA, boosts the number of mitochondria, and regulates the expression and assembly of mitochondrial respiratory chain complexes. This enhances the oxidative phosphorylation capacity of mitochondria, thereby improving their energy production efficiency [32, 64, 65]. Furthermore, PGC‐1α is involved in the regulation of mitochondrial autophagy, helping to eliminate damaged or nonfunctional mitochondria and ensuring the quality and stability of the mitochondrial population [66]. In this study, we noted an upregulation of p‐AMPK and PGC1α after OCN treatment within the hippocampus of CUMS mice. It can be seen that the antidepressant mechanism of OCN may be closely related to the AMPK/PGC1α signaling pathway. Additionally, PGC1α translocates to the nucleus, elevating the production of various antioxidant enzymes such as catalase, superoxide dismutase (SOD), and glutathione peroxidase (GPx), effectively reducing intracellular ROS levels [67, 68]. These findings provide a foundation for understanding how OCN alleviates mitochondrial injury and preserves the dynamic balance of ATP and ROS levels.

Mitochondrial fusion and fission, which are essential for maintaining the quality and functionality of mitochondria [7, 69, 70], involve multiple molecules. Among these processes, Mfn1 acts as a crucial mediator in the fusion of the outer mitochondrial membrane. Meanwhile, OPA1 and Mfn2 primarily regulate the fusion of the inner mitochondrial membrane, while FIS1 and Drp1 play major roles in mitochondrial fission [71]. Prolonged oxidative stress can disrupt these dynamic processes, ultimately compromising mitochondrial function and cell survival [72, 73]. Mitochondrial oxidative stress enhances Drp1‐mediated mitochondrial fragmentation and inhibits mitochondrial fusion [71]. This issue is also evident in the altered expression of Drp1 and Mfn2 observed in CUMS mice. PGC‐1α is also involved in modulating mitochondrial dynamics [74]; the activation of PGC‐1α leads to the upregulation of Mfn2, thereby promoting the fusion process [74, 75]. Furthermore, PGC‐1α can enhance the expression of Sestrin2, which, in turn, inhibits the mTOR signaling pathway [76]. This inhibition reduces the phosphorylation and fission activity of Drp1, leading to a decrease in mitochondrial fission. These findings support the role of OCN in maintaining mitochondrial dynamics stability by activating PGC1 in states of chronic stress. Although we detected the effects of OCN on the expression of PGC1, Mfn2, and Drp1 in this research, the results imply that OCN has the potential to maintain the stability of mitochondrial dynamics. However, the impact of OCN on the expression of other proteins related to mitochondrial fusion and fission, such as Mfn1 and FIS1, still requires in‐depth exploration in subsequent experiments to uncover its underlying mechanism of action.

Our cellular experiments using PKA or AMPK inhibitors confirmed these findings. The application of H89 or CoC significantly reduced the increase in PGC1α induced by OCN and mitigated the changes in ATP and ROS levels caused by OCN. Collectively, these findings offer substantial evidence for comprehending how OCN ameliorates depressive‐like behavior resulting from chronic stress through the AMPK/PGC1α signaling pathway.

Despite the significant findings of this study, several limitations should be acknowledged. First, our use of intraperitoneal injection of OCN, while clinically relevant, does not provide direct evidence of its site‐specific action in the hippocampus. Future studies employing hippocampal microinjection—coupled with sham control groups—will help validate the direct regulatory effects of OCN on hippocampal neurons and mitochondria. Second, our analysis of mitochondrial dynamics focused on Drp1 and Mfn2, but other key regulators such as MFN1, FIS1, and OPA1 were not evaluated. Expanded investigations into these proteins are necessary to fully elucidate OCN's impact on mitochondrial quality control networks. Third, while increasing the sample size of behavioral experiments enhanced statistical robustness, long‐term follow‐up assessments of OCN's efficacy were not included, which could provide insights into the durability of its antidepressant effects. Addressing these limitations will be critical for advancing our understanding of OCN's mechanisms. Future studies will prioritize targeted hippocampal administration, comprehensive profiling of mitochondrial proteins, and longitudinal behavioral assessments to strengthen the translational relevance of our findings.

## 5 Conclusion

In conclusion, this study confirmed the antidepressant effects of OCN in mice with depression and emphasized its role in protecting against oxidative stress and mitochondrial dysfunction caused by CUMS. We elucidated the molecular mechanism through which OCN alleviates mitochondrial injury via the AMPK/PGC1α pathway. These findings provide a novel perspective on the function of OCN in neurons, paving the way for further exploration of innovative therapeutic approaches for central nervous system disorders associated with mitochondrial dysfunction. Additionally, they offer further evidence for a better understanding of how peripheral bone tissue affects brain function.

## Conflicts of Interest

The authors declare no conflicts of interest.

## Data Availability

The data that support the findings of this study are available on request from the corresponding author. The data are not publicly available due to privacy or ethical restrictions.
